# Novel Negative Poisson’s Ratio Lattice Structures with Enhanced Stiffness and Energy Absorption Capacity

**DOI:** 10.3390/ma11071095

**Published:** 2018-06-27

**Authors:** Zeyao Chen, Zhe Wang, Shiwei Zhou, Jianwang Shao, Xian Wu

**Affiliations:** 1 School of Automotive Studies, Tongji University, Shanghai 201804, China; chenzeyao@tongji.edu.cn (Z.C.); wangzhe@tongji.edu.cn (Z.W.); shaojianwang@tongji.edu.cn (J.S.); 2 Centre for Innovative Structures and Materials, School of Engineering, RMIT University, GPO Box 2476, Melbourne 3001, Australia; shiwei.zhou@rmit.edu.au

**Keywords:** Negative Poisson’s ratio, lattice, unit cell analysis, energy absorption

## Abstract

The weak stiffness and strength of materials with negative Poisson’s ratio limits their application. In this paper, three types of novel lattices with negative Poisson’s ratio are proposed to improve not only stiffness and strength but also energy absorption capacity by embedding different ribs into a classic re-entrant structure. Unit cell analyses show these novel lattices have significantly increased Young’s modulus along the loading direction, and Type C can maintain sufficient negative Poisson’s ratio performance compared with the base lattice. In addition, the novel lattices exhibit higher yield stress, plateau stress and densification strain extracted from quasi-static compressive simulation. The lattices are prototyped by laser-based additive manufacturing and tested in quasi-static experiments, which show the experimental data match the numerical results within an error of margin. The work signifies the prospect of lattices with negative Poisson’s ratio in enhancing engineering-applicable structures, and indicates the potential of structural topology optimization in more sophisticated designs.

## 1. Introduction

Recently, due to the demand of lightweight design, cellular materials made of interconnected networks of solid struts or plates that form the edges and faces of cells [1] have been extensively studied and employed in many fields [2,3,4,5,6]. One of the most attractive merits offered by cellular materials is the high strength to weight ratio. In addition, they are also characterized with high relative stiffness, improved impact energy absorption, and thermal and acoustic insulation. Randomly structured cellular materials such as metallic and polymeric foams have been produced for a range of applications such as the middle layer of sandwich panels and automotive parts in recent decades [7]. Recently, regularly structured lattices have attracted increasing attention because the stochastic faults essentially existing in the foams can be completely avoided [8]. For instance, a two-dimensional lattice material in the shape of a honeycomb can improve the capacity of energy absorption material [9,10]. Recently, researchers found that three-dimensional (3D) lattices can improve the capacity of energy absorption for structures subjected to vibration and shock [11]. Though some initial achievements have been successful, the studies on compressive properties of lattice structures and materials in 3D are still in their infancy [12,13,14,15].

Negative Poisson’s ratio (NPR) material can contract in compression but expand in tension [16,17,18]. Such a counterintuitive auxeticity was first discovered by Lakes in 1987 and extensively studied in both theory and numerical simulation thereafter [16,17,18]. Recently, the research in auxetic material NPR was explored at molecular and atomic level and found that some face-centered cubic crystals exhibit NPR [19,20,21,22]. The exotic property of NPR is exciting and has been utilized to obtain remarkable resilience [23], improving crack resistance [24,25], increasing fracture toughness [26,27], providing higher sound absorption capacity [28] and enhancing energy absorption capability [29]. Compared with conventional materials, NPR materials show remarkable energy absorption capability [30,31,32,33,34,35,36,37,38,39,40,41,42,43,44]. Therefore, there are some attempts to use them as crashworthiness components [40,41], blasting protective structures [38,42], body armor and combat helmets [31,39] in the automobile, aerospace and defense industries. However, to our best knowledge, such applications are still in their infancy because of the compromise between NPR and a diversity of mechanical properties.

Because of the porous structures of ordinary NPR materials, their Young’s modulus and strength are inevitable weakened [23]. To improve the in-plane stiffness of the normal re-entrant hexagonal honeycomb, a splined-reentrant honeycomb and a stiffened-reentrant honeycomb were conceived recently [45]. Recently, a NPR structure was engineered to improve stiffness by adding a narrow straight rib into the unit cell of a classical re-entrant structure [46]. To enhance the in-plane mechanical properties, a honeycomb was reconstructed by embedding the rhombic configuration into the normal re-entrant hexagonal honeycomb [47]. A 3D version of an 2D auxetic material was proposed by adding some narrow ribs into all cells and its improved elastic behavior and dependence on the geometric parameters were systematically investigated by theoretical and numerical analysis [48]. Two types of new 2D re-entrant topologies were proposed with enhanced auxetic responses constructed by adding sinusoidal-shaped ribs and extra vertical ribs into classical re-entrant topology [49]. The abovementioned work has made some achievements in maintaining the coexistence of NPR and good mechanical properties, but more work is still needed.

In this paper, we propose three 3D NPR lattices (Type B, Type C and Type D) by embedding different ribs into the conventional re-entrant cell (Type A) to improve the stiffness and capacity of energy absorption. We noted Young’s modulus and Poisson’s ratio of cell can be adjusted by length ratio and height ratio. To evaluate the enhanced effect for stiffness, parametric studies of mechanical properties are performed on these novel NPR cells respectively. The novel cells can improve Young’s modulus and the Type C can maintain obvious NPR performance compared with the conventional lattice (Type A). In addition, quasi-static compression analyses of four NPR lattices are conducted utilizing non-linear finite element simulation to study the energy absorption capability of these structures. Type C shows large plateau stress, stiffness and strength similar to Type B and Type D, but with lower relative density and wider plateau region. Furthermore, the quasi-static experiment results of novel NPR samples which are fabricated by selective laser manufacturing are employed to validate the simulation model. These novel NPR structures introduced here could provide new topology designs and new potential for energy absorption structure.

## 2. Novel Types of NPR Structure

**Figure 1:**
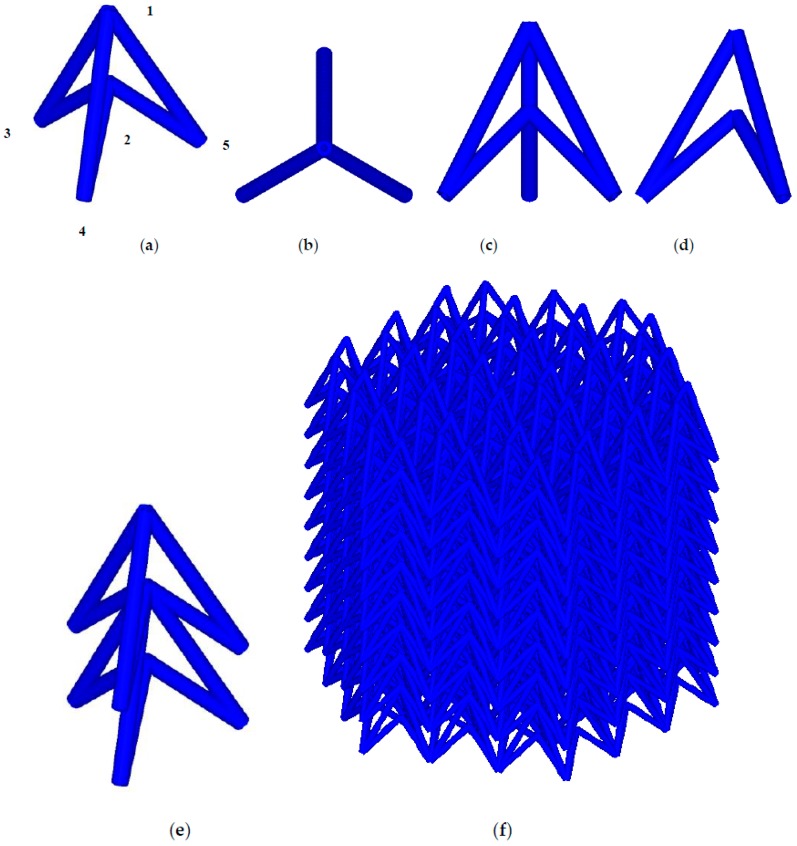
The schematic of Type A lattice in: (**a**) 3D viewpoint; (**b**) top view (**c**) front view; (**d**) left view; and (**e**) an array.

**Figure 2:**
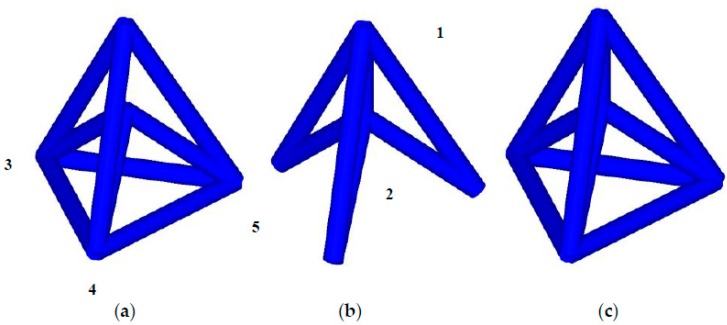
The schematic of a lattice in the shape of (**a**) Type B; (**b**) Type C; and (**c**) Type D.

Figure 1 illustrates the unit cell of a conventional NPR lattice (Type A) in different viewing perspective and its structure when the cells are periodically ranked in space. Type A is designed in this study based on re-entrant mechanism which is known to be one main mechanism of NPR material, firstly proposed by Evans [50]. Based on Type A, we develop other three types of NPR cell, called Type B, Type C and Type D respectively, to improve the mechanical properties of the novel NPR structure by adding ribs in different positions, shown in Figure 2. Type B is built by adding three ribs between the point 3-4-5, The Type C cell is constructed by embedding a rib between point 1 and point 2, and Type D is established by overlapping Type B and Type C.

**Figure 3:**
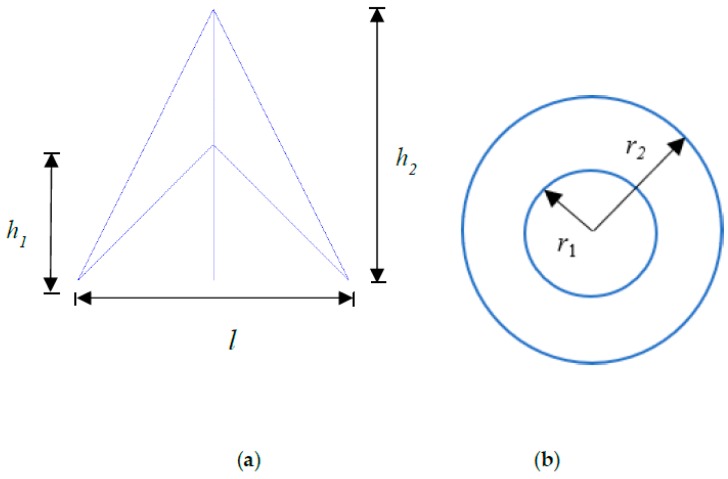
Cell geometry size: (**a**) topology shape size; (**b**) beam size.

As shown in Figure 3, the topology of unit cells can be characterized using three geometrical parameters: height parameter $h_{1}$, height parameter $h_{2}$ and length parameter *l*. In addition, inner radius $r_{1}$ and outer radius $r_{2}$ are utilized to describe the section of beam because a circular tube or cylinder is considered to be the beam type for these lattice cells. Here, we define two shape parameters: $\alpha_{h}=\frac{h_{1}}{h_{2}}$ and $\alpha_{l}=\frac{l}{h_{2}}$. In this paper, the scale parameter is $h_{2}$, the shape parameters are the height ratio $\alpha_{h}$ and length ratio $\alpha_{l}$, the beam parameters are outer radius $r_{1}$ and inner radius $r_{2}$. Parameters:${\;\alpha }_{h}$, ${\;\alpha }_{l}$, $\;h_{2}$*,* $r_{1}$, $r_{2}$ are used to define geometries of these cells.

Similar to other cellular materials, the mechanical properties of the NPR lattice structures are also closely related to its relative density rather than other features [51]. Therefore, relative density is significant for analyzing the mechanical properties of the cellular structure. The formula of the relative density for cellular material is given by [52]:

(1) $$\rho =\frac{\rho_{t}}{\rho_{f}},$$

where $\rho_{t}$ represents measured density of cellular structure, $\rho_{f}$ denotes the density of the base material that fabricates the cellular structure. Using the unit cell analysis method, the relative density calculation is given as following [52]:

(2) $$\rho =\frac{V_{t}}{V},$$

where $V_{t}$ is volume of solid in cellular material, *V* is the spatial volume occupied by the unit cell. Then the relative density formulas for the four types of designed NPR structures are derived by unit cell analyses, shown in Table 1.

## 3. Unit Cell Analyses of NPR Structures

### 3.1. Young’s Modulus and Poisson’s Ratio of Unit Cell

**Figure 4:**
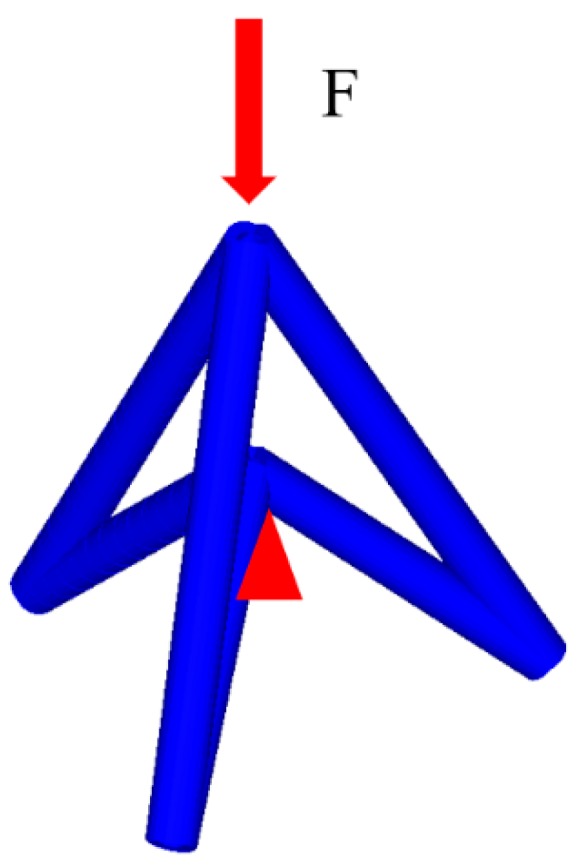
The model of unit cell analysis.

To analyze the equivalent mechanical properties of lattice structures, the unit cell analysis method is generally adopted [53]. The representative volume elements of these NPR lattice structures are shown in Figure 1a and Figure 2. The unit cell analysis of NPR structures is set as shown in Figure 4. We consider the Young’s modulus and Poisson’s ratio in principal loading direction (*z*-axis direction) in this paper. Formulas of the effective Young’s modulus* E *and Poisson’s ratio *v *are given:

(3) $$E_{e}=\frac{F}{A\left(\Delta L/L\right)},$$

(4) $$\upsilon_{e}=-\frac{\left(\Delta u/u\right)}{\left(\Delta L/L\right)},$$

where *A* is the projected area of the unit cell in the loading direction (Z direction), *L* is the length of junction between upper and lower unit cell and *u* is the radial length. The unit cell analyses presented here are similar to spatial beams considering bending and torsion. In this paper, we use the Timoshenko beam theory to solve the unit cell analysis model [53]. The Timoshenko beam takes into account shear deformation and rotational bending effects, making it suitable for describing the behavior of thick spatial beams. Elastic Timoshenko beams are used to build the unit cells under small elastic deformation. These finite element analyses (FEA) of unit cells are carried out using RADIOSS (Altair Engineering Inc., Troy, MI, USA).

Aluminum alloy whose Young’s modulus is 70 GPa and Poisson’s ratio is 0.27 is adopted as base material in the computations. To identify how geometrical parameters and enhanced pattern affect the effective mechanical properties of structures designed in Section 2, shape parametric studies for four NPR structures have been conducted using the unit cell models demonstrated here. The numerical results are presented and discussed in following sections. Comparison of these four NPR structure cell analyses is employed to reveal enhancement effect of cell topology.

### 3.2. Type A Cell Parametric Studies

**Figure 5:**
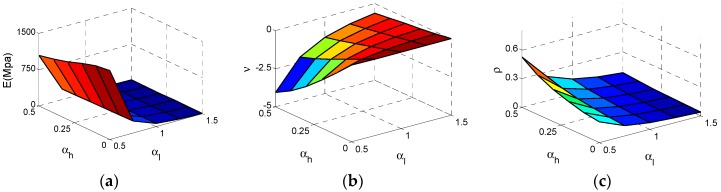
The Surfaces of effective properties for Type A cell: (**a**) effective Young’s modulus *E*; (**b**) effective Poisson ratio *v*; (**c**) relative density  ρ.

**Figure 6:**
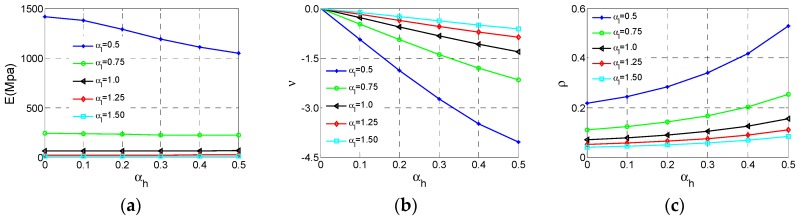
The changes of the effective properties with respect to the $\alpha_{h\;}$ and $\alpha_{l\;}$ for Type A cell: (**a**) effective Young’s modulus *E*; (**b**) effective Poisson ratio *v*; (**c**) relative density  ρ.

Figure 5 shows how the effective Young’s modulus *E*, Poisson’s ratio *v* and the relative density ρ of NPR Type A vary with $\alpha_{h\;}$ and $\alpha_{l\;}$. In this section, and following section, we just consider $\alpha_{h\;}\;$ and $\;\alpha_{l\;}$ parametric studies at $h_{2}$ = 20 mm, $r_{1}$ = 0.5 mm, $r_{2}$ = 1 mm. The numerical values of *E* and *v *are obtained from Equations (3) to (4) after each unit cell model is analyzed. The values of ρ are displayed in Figure 5 and Figure 6, which are extracted from the relative density formula for Type A cell listed in Table 1.

As shown in Figure 6a, the effective Young’s modulus* E *reaches the maximum value at $\alpha_{h\;}$ = 0, $\alpha_{l\;}$= 0.5 and the effective *E* increases substantially with the $\alpha_{l\;}$ decrease. In contrast, the influence of $\alpha_{h\;}$ is tiny. In Figure 6b, the effective Poisson ratio *v* reaches the minimum value −4 at $\alpha_{h\;}$ = 0.5, $\alpha_{l\;}$ = 0.5 and both $\alpha_{l\;}$ and $\alpha_{h\;}$ have important impacts on Poisson’s ratio. With the increase of $\;\alpha_{h\;}$ or decline of $\alpha_{l\;}$, the *v* declines significantly. For relative density ρ, the law of varying is very clear. In Figure 6c, ρ reaches the maximum value at $\alpha_{h\;}$ = 0.5, $\alpha_{l\;}$ = 0.5. With increase of $\alpha_{h\;}$ or decline of $\alpha_{l\;}$, ρ increases.

The relationship between Young’s modulus and relative density is very important for lattice material because we are not only looking for lighter material or structure but also with sufficient stiffness and strength. How Young’s modulus and strength vary with relative density has attracted more attention. For cellular materials, many mechanical properties including stiffness and strength always scale with the relative density. According to the results derived by Gibson and Ashby [1], the dependence of effective Young’s modulus on relative density of the cellular structure can be expressed in the following form:

(5) $$E=a{\bar{\rho }}^{e}.$$

**Figure 7:**
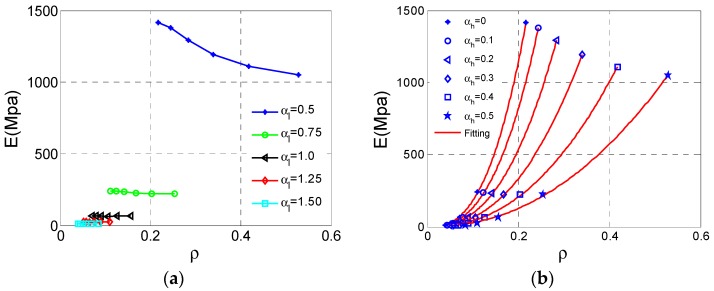
The changes of effective Young’s modulus *E* for Type A cell with respect to the relative density ρ: (**a**) data set; (**b**) fitting curves.

In the formula, the exponent *e* denotes the stiffness property of this type of cellular material. The Young’s modulus and relative density data are gathered shown in Figure 7a. It is shown that these data are not consistent with the exponent law if we consider the variation of $\alpha_{l\;}$. Therefore, it is necessary to fit these data respectively for separated $\alpha_{l\;}$ plotted in Figure 7b. To evaluate the fitting accuracy of the exponent equation, the indicator namely R-square (*R*^2^) is defined as

(6) $$R^{2}=1-\frac{\sum_{j=1}^{m}{\left(y_{j}-{\hat{y}}_{j}\right)}^{2}}{\sum_{j=1}^{m}{\left(y_{j}-{\bar{y}}_{j}\right)}^{2}}.$$

If the *R*^2^ is over 0.9, fitting is considered to be valid. The exponents and *R*^2^ values can be derived from the fitting shown in Table 2. As shown in these results, all six fitting curves meet the fitting accuracy requirement.

### 3.3. Type B Cell Parametric Studies

**Figure 8:**
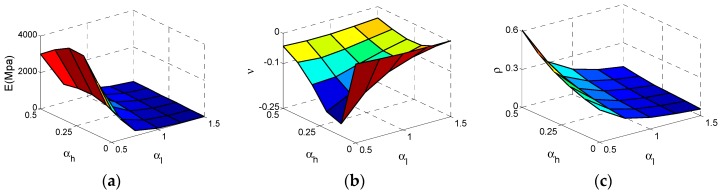
The Surfaces of effective properties for Type B cell: (**a**) effective Young’s modulus *E*; (**b**) effective Poisson ratio *v*; (**c**) relative density ρ.

**Figure 9:**
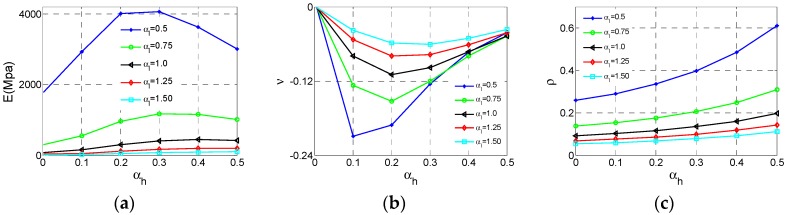
The changes of the effective properties with respect to the height ratio $\alpha_{h\;}$ and the length ratio $\alpha_{l\;}$ for Type B cell: (**a**) effective Young’s modulus *E*; (**b**) effective Poisson ratio *v*; (**c**) relative density  ρ.

These results and data of Type B unit cell analyses are collected shown in Figure 8. As can be seen from Figure 9a, the effective Young’s modulus *E* reaches the maximum value at $\alpha_{h\;}$ = 0.3, $\alpha_{l\;}$ = 0.5 and the effective *E* increases substantially with the $\alpha_{l\;}$ decrease. In contrast, the influence of $\alpha_{h\;}$ is not regular, and we can get large *E* at appropriate $\alpha_{h\;}$. In Figure 9b, the effective Poisson ratio *v* reaches the minimum value −0.21 at $\alpha_{h\;}$ = 0.1, $\alpha_{l\;}$ = 0.5 and both $\alpha_{l\;}$ and $\alpha_{h\;}$ have insignificant effect on Poisson’s ratio *v*. The loss of NPR behaviors for the Type B cell can be observed from the Poisson’s ratio *v* close to zero. For relative density ρ of the Type B cell, the law of change is very clear and similar to Type A shown in Figure 9c.

**Figure 10:**
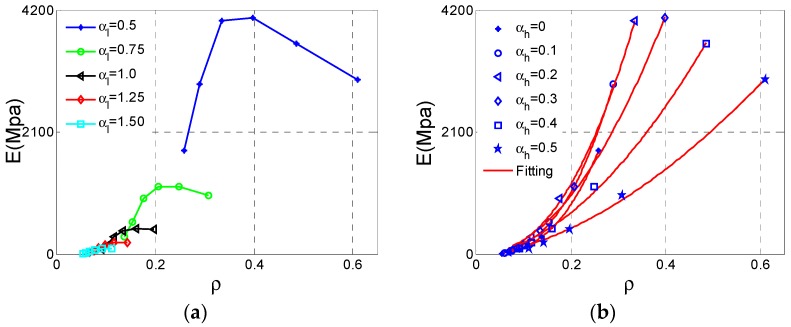
The changes of effective Young’s modulus *E* for Type B cell with respect to the relative density  ρ: (**a**) data set; (**b**) fitting curves.

The relationship of Young’s modulus *E* and relative density ρ of Type B are analyzed utilizing data shown in Figure 10a. The fitting method in group style for different $\alpha_{l\;}$ as the Type A also be adopted to analysis these data, displayed in Figure 10b. These fitting parameters are demonstrated in Table 3, which shows all fitting curves are valid.

### 3.4. Type C Cell Parametric Studies

**Figure 11:**
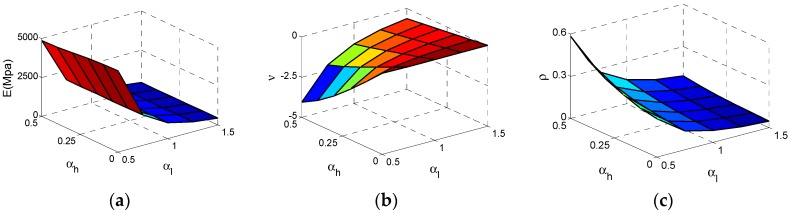
The Surfaces of effective properties for Type C cell: (**a**) effective Young’s modulus *E*; (**b**) effective Poisson ratio *v*; (**c**) relative density  ρ.

**Figure 12:**
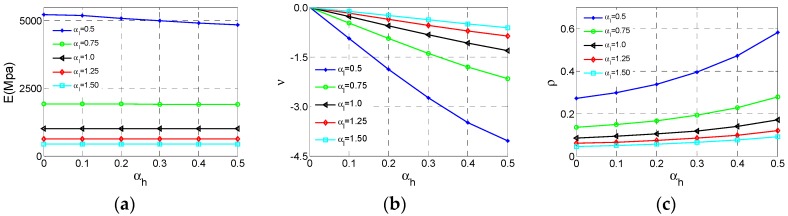
The changes of the effective properties with respect to the height ratio $\alpha_{h\;}$ and the length ratio $\alpha_{l\;}$ for Type C cell: (**a**) effective Young’s modulus *E*; (**b**) effective Poisson ratio *v*; (**c**) relative density  ρ.

After we conducted calculations on the Type C cell, these results and data are shown in Figure 11. As can be seen from Figure 12a, the effective Young’s modulus *E* reaches the maximum value at $\alpha_{h\;}$ = 0, $\alpha_{l\;}$ = 0.5 and the effective *E* increases substantially with the decrease of $\alpha_{l\;}$. In contrast, the influence of $\alpha_{h\;}$ for *E* is sufficiently less that it can be ignored. In Figure 12b, the effective Poisson ratio *v* reaches the minimum value −4 at $\alpha_{h\;}$ = 0.5, $\alpha_{l\;}$ = 0.5 and both $\alpha_{l\;}$ and $\alpha_{h\;}$ have important impact on Poisson’s ratio which is similar to the Type A cell. For relative density  ρ of the Type C cell, the law of change is very clear and similar to Type A. In Figure 12 c, ρ reaches the maximum value at $\alpha_{h\;}$ = 0.5, $\alpha_{l\;}$ = 0.5. With the increase of $\alpha_{h\;}$ or decline of $\alpha_{l\;}$, the relative density ρ increases.

**Figure 13:**
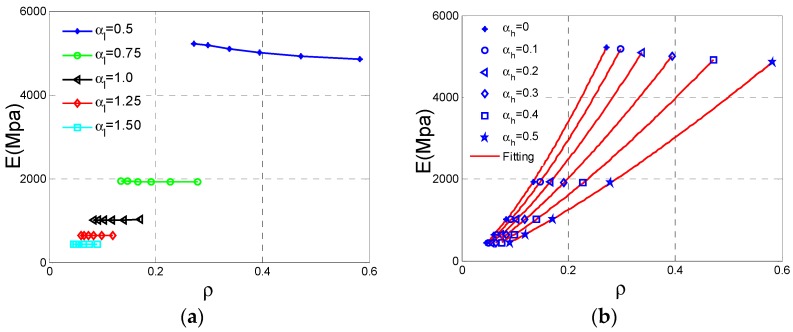
The changes of effective Young’s modulus *E* for Type C cell with respect to the relative density  ρ: (**a**) data set; (**b**) fitting curves.

The effective Young’s modulus and relative density data of Type C are plotted in Figure 13a. As we can see, these data are not consistent with the exponent law considering the variation of $\alpha_{l\;}$. Therefore, it is necessary to fit these data by separating $\alpha_{l}$ shown in Figure 13b. These fitting parameters are demonstrated in Table 4. All *R*^2^ are over 0.9 in Table 4, which means all these fitting curves are valid.

### 3.5. Type D Cell Parametric Studies

**Figure 14:**
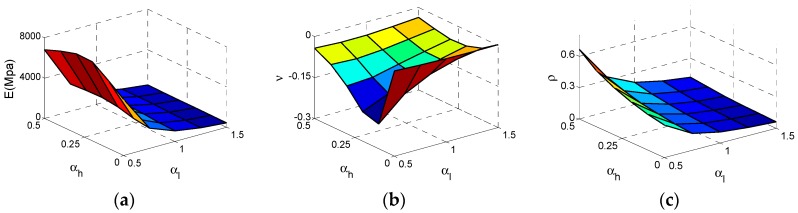
The Surfaces of effective properties for Type D cell: (**a**) effective Young’s modulus *E*; (**b**) effective Poisson ratio *v*; (**c**) relative density  ρ.

**Figure 15:**
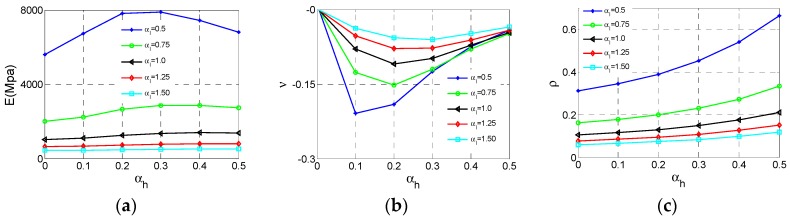
The changes of the effective properties with respect to the height ratio $\alpha_{h\;}$ and the length ratio $\alpha_{l\;}$ for Type D cell: (**a**) effective Young’s modulus *E*; (**b**) effective Poisson ratio *v*; (**c**) relative density ρ.

After the Type D cell analyses are carried out, these results and data are displayed in Figure 14. As can be seen from Figure 15a, the effective Young’s modulus *E* reaches maximum at $\alpha_{h\;}$ = 0.3, $\alpha_{l}$ = 0.5 and the effective *E* increases substantially with the $\alpha_{l}\;$ decrease. In contrast, the influence of $\alpha_{h\;}$ is not regular and we can get large effective* E *at appropriate $\alpha_{h\;}$ which are very similar to Type B. In Figure 15b, the effective Poisson ratio *v* reaches minimum value −0.21 at $\alpha_{h\;}$ = 0.1, $\alpha_{l}$ = 0.5 and both $\alpha_{l}$ and $\alpha_{h\;}$ have unimportant impacts on Poisson’s ratio as *v *is close to 0, which is very similar to the Type B cell. For relative density ρ of the Type D cell, the law of varying is very clear and similar to other above type. In Figure 15c, ρ reaches the maximum value at $\alpha_{h\;}$ = 0.5, $\alpha_{l}$ = 0.5. With the increase of $\alpha_{h\;}$ or decline of $\alpha_{l}$, the ρ increases.

**Figure 16:**
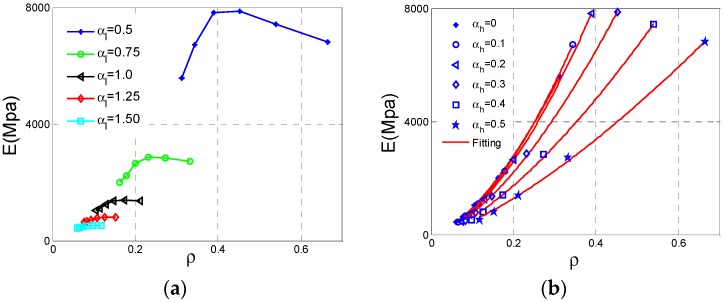
The changes of effective Young’s modulus *E* for Type D cell with respect to the relative density ρ: (**a**) data set; (**b**) fitting curves.

The elastic modulus and relative density data are put together in Figure 16a. As we can see, these data are not consistent with the exponent law if we consider the $\alpha_{l}$ variation. Therefore, it is necessary to fit these data by separating $\alpha_{l}$ shown in Figure 16b. These fitting parameters are demonstrated in Table 5, which means all these fitting curves are valid.

### 3.6. Comparison of Exponent

**Figure 17:**
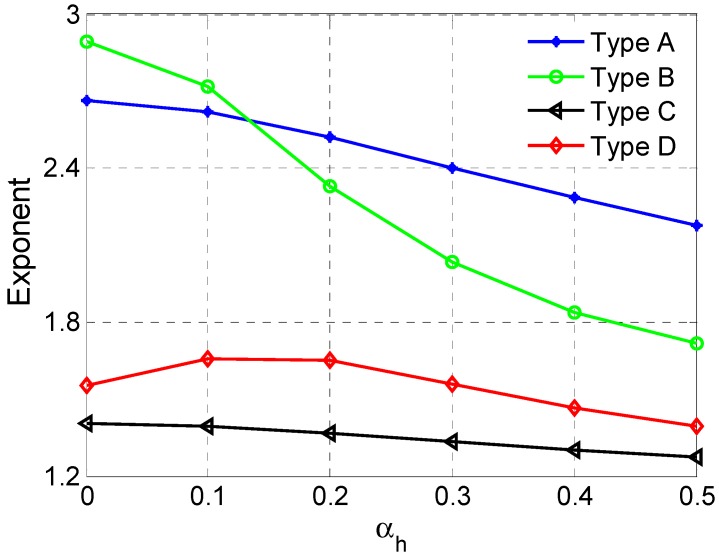
The dependence of the exponent *e* on the height ratio.

Because the relationship between the Young’s modulus and relative density obeys the exponential law, it is reasonable to use the exponent *e* to measure the enhancement of stiffness for the lattices. In case a large exponent, the Young’s modulus will reduce sharply with decrease of relative density. Therefore, small exponents stand for high stiffness enhanced efficiency. Figure 17 plots the exponents of four types of cell at $\alpha_{h}$ range from 0 to 0.5. As can be seen from Figure 17, the exponents of enhanced cells are significantly reduced. The Type C cell always has the lowest exponents at different $\alpha_{h}$, which means the Type C cell has the highest enhanced efficiency in the loading direction. The above-mentioned means the Type C structure can not only maintain superior NPR property but also enhance the stiffness.

## 4. Quasi-Static Analyses of NPR Structure

### 4.1. NPR Structure and Quasi-Static FE

**Figure 18:**
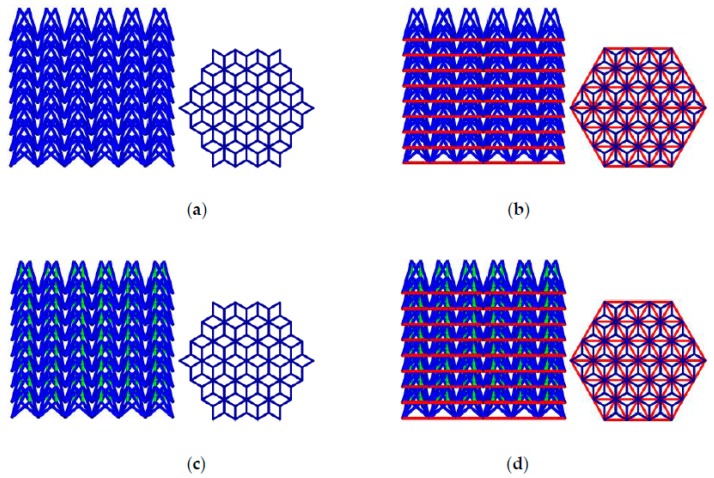
Structures fabricated by four novel NPR cells: (**a**) Type A NPR structure; (**b**) Type B NPR structure; (**c**) Type C NPR structure; (**d**) **T**ype D NPR structure.

Four NPR lattice structures are fabricated as 54 cells per layer and 9 layers using these novel NPR cells are shown in Figure 18. These structures’ section is hexagon with 60mm sides. All effective lengths of these structures are 100 mm. These four NPR structures’ mass are 0.356, 0.459, 0.387, 0.490 kg respectively. These four NPR structures’ relative density are 0.1444, 0.1861, 0.1569, 0.1987 respectively.

To study the compressive mechanical properties of the designed structures, non-linear FEA are conducted. The nonlinear FEA program LS-DYNA (Livermore Software Technology Corporation, Livermore, CA, USA) is used to simulate the compressive response mechanism of NPR lattice structures. Due to the fact that continuum solid elements are computationally expensive and complicated, plastic beam elements with large deformation are used to simulate NPR lattice structures. Some papers [12,54] have verified that beam elements can obtain accuracy as solid element and improve computation efficiency remarkably.

The Belytschko-Schwer resultant beam element is excellent for simulating beam plastic bending, compression-stretching and torsion [55]. The Johnson-Cook empirical constitutive equation for MAT-98 material model [55] is appropriate for the Belytschko-Schwer beam element material, regardless of the effect of temperature, as shown below

(7) $$\sigma =\left(A+B\varepsilon^{n}\right)\left(1+C\ln\frac{\dot{\varepsilon }}{{\dot{\varepsilon }}_{0}}\right).$$

The parameters are: *A* = 448 MPa; *B* = 343 MPa; *C* = 0.01; *n* = 0.41 for the aluminum alloy 7075 used in this research. The impactor is modeled by rigid components whose material model is MAT-20.

**Figure 19:**
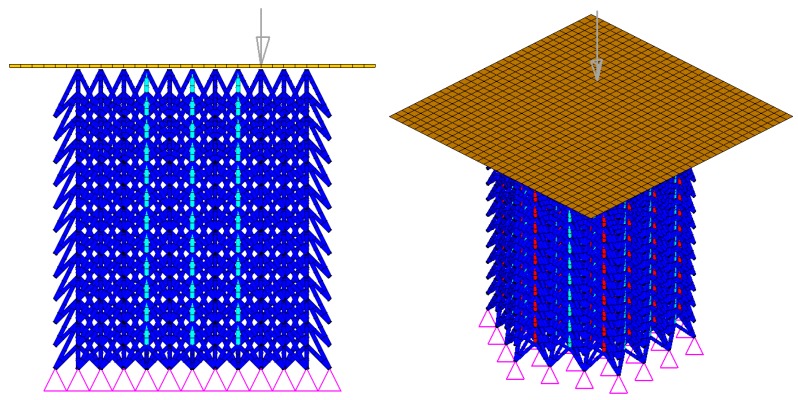
The FE model of NPR lattice structure.

In the non-linear FEA simulations, forces between parts are transferred with contact algorithms. The so-called one-way contact algorithm (*CONTACT_AUTOMATIC_NODES_TO_SURFACE) is used to simulate contact between impactor and lattice, in which only slave nodes (lattice nodes) are checked for penetration of the master segments (rigid impactor). Self-contact of the lattice structure is modelled using a beam-to-beam contact algorithm (*CONTACT_AUTOMATIC_GENERAL), which is effective for defining the edge-to-edge contact for beam elements. Static and dynamic friction coefficients are defined as 0.20 for all contact cases. The rigid plate with prescribed velocity is used to model rigid indenter. The LS-DYNA FE model is established with conditions, as shown in Figure 19.

### 4.2. FE Model Validation

To validate the FE model of NPR lattice structure, quasi-static compression experiment is carried out. First of all, NPR lattice structure (Type C, 24 cells per layer, 4 layers, $\alpha_{h}$ = 0.5, $\alpha_{l}$ = 1, *h*_2_ = 20 mm, *r*_1_ = 0 mm, *r*_2_ = 0.8 mm) is manufactured by selective laser melting (SLM) which is one of the main additive manufacturing techniques. The SLM 250 HL (SLM Solutions Group AG, Lübeck, SH, Germany) was used to prototype these lattices by printing AlSi10Mg powder. This powder’s thermal conductivity is 150 W/mK at 20 °C. This raw material is in spherical shape with a diameter ranging from 20 μm to 63 μm. The SLM equipment has a power of 100 W and a speed of 300 mm/s, which allows 80 μm hatch spacing, 30 μm layer thickness and ~99.5% compact density.

**Figure 20:**
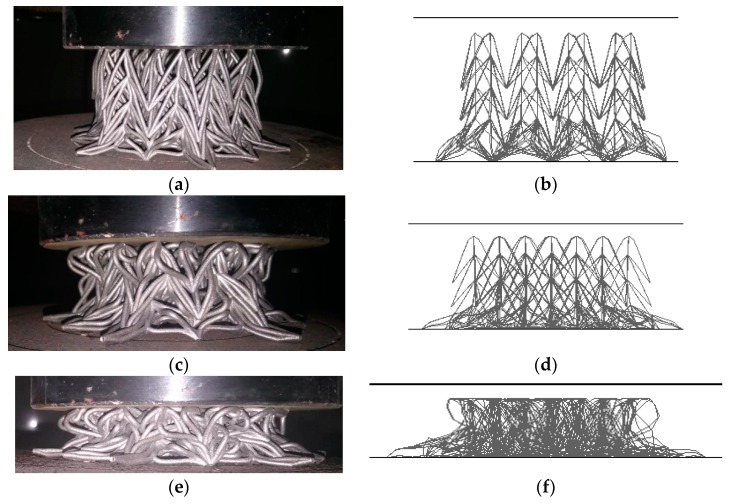
Comparison of compressive deformation process: (**a**) experiment, 10 mm deformation; (**b**) simulation, 10 mm; (**c**) experiment, 20 mm; (**d**) simulation, 20 mm; (**e**) experiment, 30 mm; (**f**) simulation, 30 mm.

**Figure 21:**
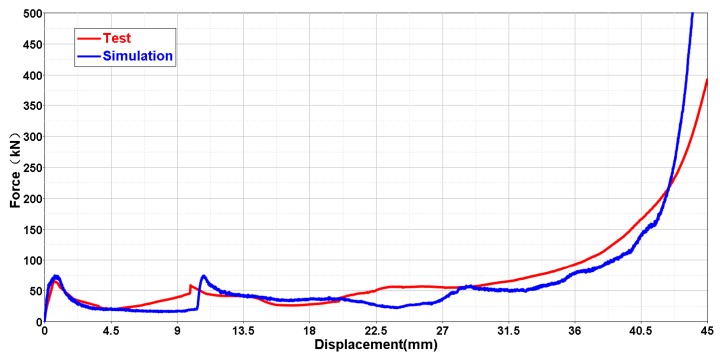
Comparison of the force-displacement curves of the NPR structure (Type C, 24 cells per layer, 4 layers, $\alpha_{h}$ = 0.5, $\alpha_{l}$ = 1, *h*_2_ = 20 mm, *r*_1_ = 0 mm, *r*_2_ = 0.8 mm).

Figure 20 shows the compressive deformation comparison between experiment and numerical simulation. There is good agreement between experiment and numerical simulation. Both samples in experiment and simulation show obvious NPR effects in the compressive process. The force-displacement curve of the experiment is used to further validate the FE model, plotted in Figure 21. Although some details are different between the experiment and the simulation, the overall trends of these two curves are similar. In particular, the simulation result has good agreement with the experiment on the early stage of compression.

### 4.3. Quasi-Static Analysis Results of the NPR Structure

**Figure 22:**
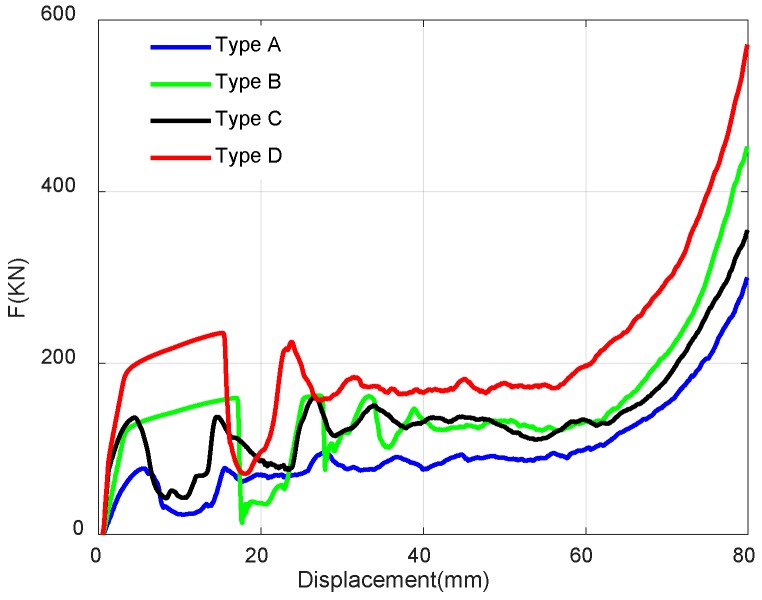
Comparison of four lattices in terms of the quasi-static compressive force and displacement.

**Figure 23:**
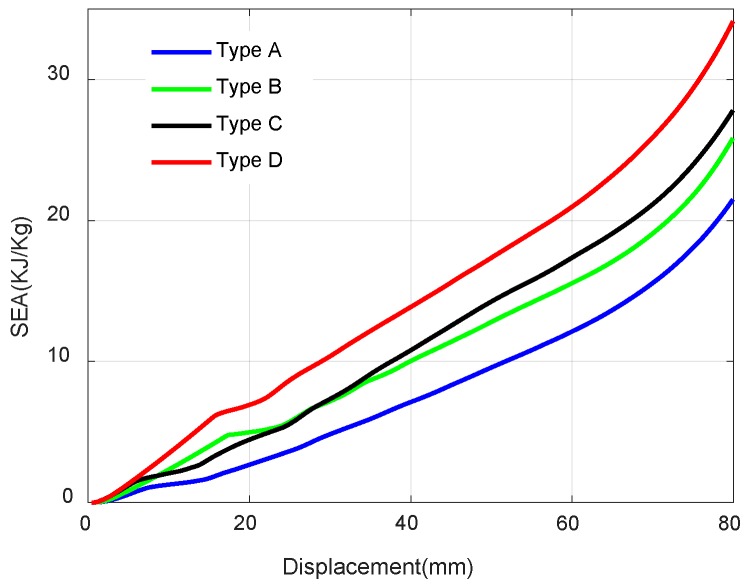
Comparison of four lattices in terms of quasi-static compressive SEA and displacement.

First of all, we extract the reaction force from the simulation results as compressive load. The force-displacement curves of four NPR lattice structures can be obtained respectively shown in Figure 22, which shows that the enhanced structures have reinforced initial stiffness and higher compressive force. Furthermore, specific energy absorption (SEA=Energy/Mass) is employed to characterize the energy absorption capacity for structure. The compressive energy absorption can be calculated by $\overset{D}{\underset{0}{\int }}Fds$. Therefore, the SEA-Displacement curves of NPR lattices are plotted in Figure 23. The novel NPR structures have higher SEA compared to the Type A structure, which exhibits improved energy absorption capacity.

To describe the quasi-static compressive performance more appropriately, the nominal stress and strain are defined as follows:

(8) $$\begin{array}{l} \sigma =\frac{F}{A_{s}},\\ \varepsilon =\frac{\Delta L_{s}}{L_{s}}. \end{array}$$

Here $A_{s}$ denotes sectional area of the sample, $L_{s}\;$ is original length of the sample in the compressive direction.

**Figure 24:**
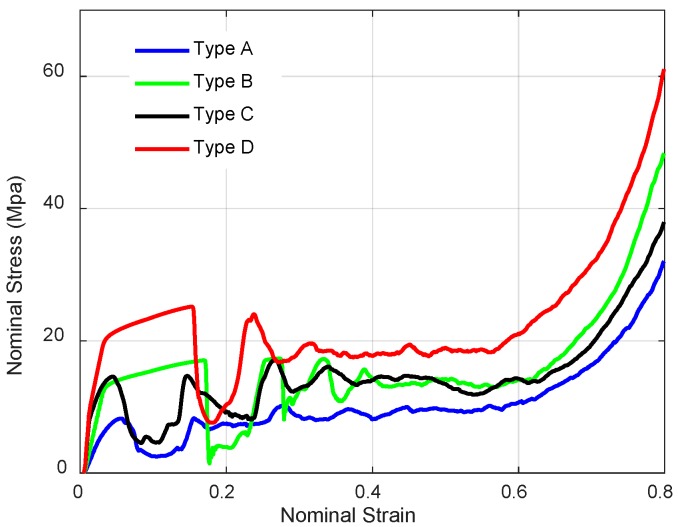
Nominal stress-strain curves of NPR lattice structures.

**Figure 25:**
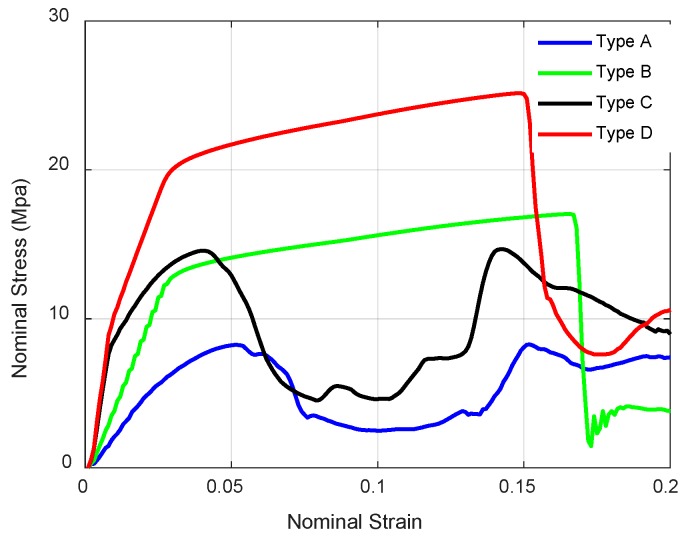
Nominal stress-strain curves of NPR lattice structures (strain range from 0 to 0.2).

These nominal stress-strain curves of four NPR structures are demonstrated in Figure 24. These curves show the classic three regimes: the elastic deformation regime; the plateau regime; and the densification regime. The obvious wide plateau region can be found in these stress-strain curves. After the strain exceeds the plateau region, the stress increases rapidly. The elastic deformation regime can be observed clearly in Figure 25. The yield stress and elastic modulus can be obtained easily from the nominal stress-strain curves which are presented in Table 6. Here, the first elastic modulus is used to characterize the initial stiffness and the first stress peak determines the yield stress, which describes the strength. The first elastic moduli of novel NPR structures, especially for Type C and Type D, are higher than the value for Type A, which means the enhanced structures significantly improved stiffness. Furthermore, it is shown that the enhanced structures’ yield stresses are larger than Type A, which means the novel NPR structures can reinforce the strength.

To identify the energy absorption capability of material, the energy efficiency [56] is determined by:

(9) $$\eta =\frac{\int_{0}^{\varepsilon_{m}}\sigma d\varepsilon }{\sigma_{m}}.$$

**Figure 26:**
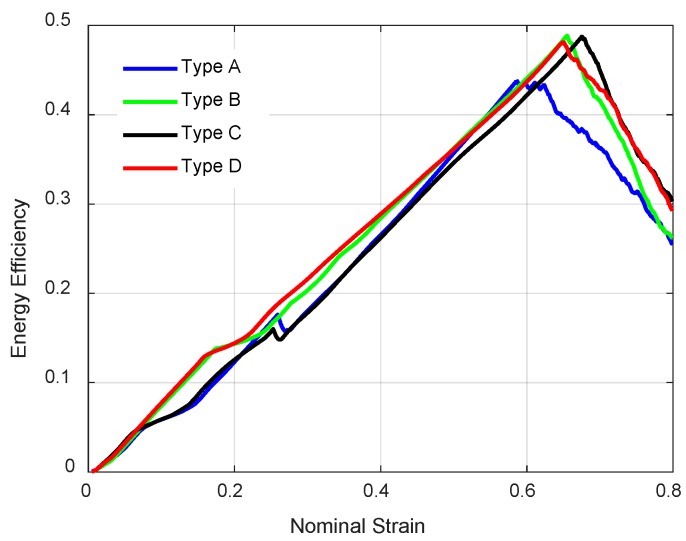
Energy efficiency curves of NPR lattice structures.

This formula is used to calculate the energy efficiency η when the strain is $\varepsilon_{m}$. The compressive stress-strain curves of these four structures are obtained shown in Figure 26. The energy efficiency determines a representative densification strain used in paper [56] as:

(10) $${\frac{d\eta }{d\varepsilon }|}_{\varepsilon =\varepsilon_{d}}=0.$$

When the energy absorption efficiency reaches the global maximum point on the energy efficiency-strain curve, we define the corresponding strain $\varepsilon_{d}$ as densification strain. Therefore, we can easily figure out the densification strains from the energy efficiency-strain curves shown in Figure 26.

The plateau stress is another significant indicator to evaluate energy absorption capability of cellular materials, which is determined by:

(11) $$\sigma_{pl}=\frac{1}{\varepsilon_{d}-\varepsilon_{y}}\int_{\varepsilon_{y}}^{\varepsilon_{d}}\sigma d\varepsilon .$$

The plateau region starts from the yield strain $\varepsilon_{y}$ where the stress reaches to the first peak of stress-strain curve. The plateau region ends at densification strain $\varepsilon_{d}$. The plateau stress refers to the average stress in the plateau region. Table 7 provides the densification strain and plateau stress of four NPR lattice structures quasi-static compression. It can be seen that enhanced structures can increase the densification strain and plateau stress, which indicates improving energy absorption capability. In addition, the Type C structure has the largest densification strain, which means Type C has the widest plateau regime. For the plateau stress, Type D has the largest value 18.76 MPa; Type B and Type C have similar values at 13 MPa.

**Figure 27:**
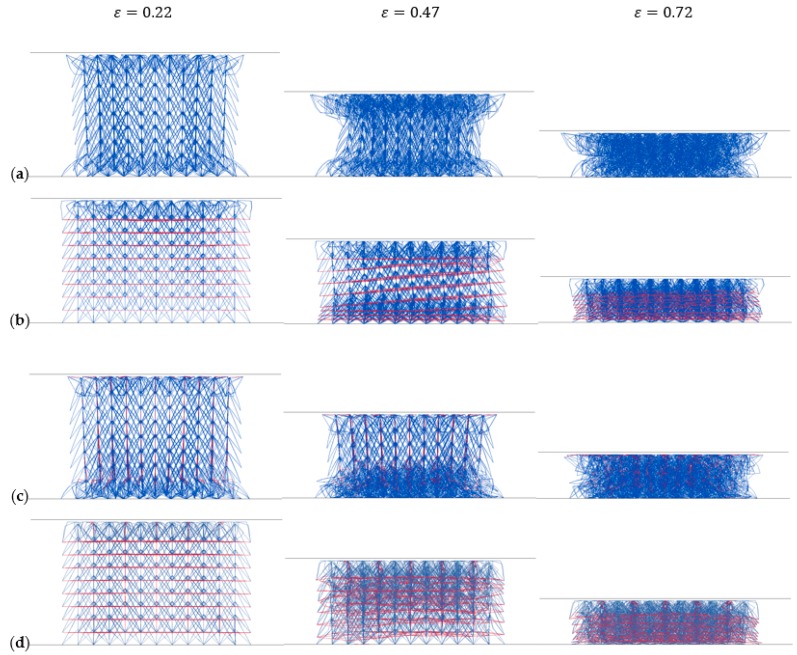
Compressive deformation of four NPR lattice structures: (**a**) Type A NPR lattice; (**b**) Type B NPR lattice; (**c**) Type C NPR lattice; (**d**) Type D NPR lattice.

Figure 27 plots the compressive deformation processes of four NPR structures. Type B and Type D display layer-by-layer collapse pattern. Type A and Type C show different collapse patterns. There are obvious NPR effects appearing in Type A and Type C compression. Nevertheless, the lateral change of Type B and Type D lattices in compression is neglectable, which means they present zero Poisson’s ratio. These phenomena coincide with the cell Poisson’s ratio analysis results.

## 5. Discussion and Conclusions

By embedding some ribs into classic re-entrant cell, three types of lattice structures are proposed to improve mechanical properties. Unit cell analysis results show these lattices can significantly improve Young’s modulus. In particular, the Type C lattice validates the coexistence of negative Poisson’s ratio and strengthened stiffness.

The quasi-static response of these lattices is simulated via finite element analysis with the Belytschko-Schwer beam element in LS-DYNA and the results match the experimental data within a small margin of error. From the nominal stress-strain curves, it is possible to figure out the elastic modulus, yield stress, plateau stress and densification strain easily. The work reveals that lattice structures can remarkably reinforce the stiffness and strength, evidently improve the energy absorption capability, and significantly increasing the densification strain and plateau stress while maintaining the Poisson’s ratio below or close to zero. In conclusion, this paper opens a window to utilize the uncommon Poisson’s ratio residing in negative or zero territory for lattice structures.

The specimen used in this paper was prototyped by the SLM technique. Thanks to the rapidly developed additive manufacturing of SLM [57], the complicated lattices, especially the type of re-entrant, can be easily fabricated in high resolution. Over the last decade, the SLM technique has gained wide acceptance due to the continuously improving process parameters (smaller layer thickness, smaller powder size), resulting in excellent mechanical properties and low porosity (down to 0.1%). Furthermore, the SLM technique shows the advantage of being able to process a wide range of metallic materials such as steel alloy, aluminum alloy, titanium alloy, ceramic, and cooper. It can be expected that there are more and more sophisticated materials and structures such as lattices that can be manufactured by additive manufacturing techniques such as SLM.

The buckling and the deformation in plastic stage are not considered in this paper. However, in future, both the elastic-plastic properties and the collapse surface resulting from buckling will be considered in the unit cell analysis to predict the strength. Because a small fraction of the lattices fracture in the quasi-static compressive experiment, evaluation of these fractures should be undertaken in the future. Moreover, the dynamically impacting properties at different velocities should be explored in the next step to evaluate the crashworthiness of these lattices. For these enhanced properties mentioned above, any presented investigation may provide novel concepts for the optimization and design of NPR materials and structures.

## Figures and Tables

**Table 1:** The formula of relative density for four types of NPR lattices.

Types of NPR Cells	Relative Density
Type A	$\rho =\frac{4\sqrt{3}\pi \left(\sqrt{1+\alpha_{l}^{2}/3}+\sqrt{\alpha_{h}^{2}+\alpha_{l}^{2}/3}\right)\left(r_{2}^{2}-r_{1}^{2}\right)}{\left(1-\alpha_{h}\right){\left(\alpha_{l}h_{2}\right)}^{2}}$
Type B	$\rho =\frac{4\sqrt{3}\pi \left(\sqrt{1+\alpha_{l}^{2}/3}+\sqrt{\alpha_{h}^{2}+\alpha_{l}^{2}/3}+\alpha_{l}/2\right)\left(r_{2}^{2}-r_{1}^{2}\right)}{\left(1-\alpha_{h}\right){\left(\alpha_{l}h_{2}\right)}^{2}}$
Type C	$\rho =\frac{4\sqrt{3}\pi \left[\sqrt{1+\alpha_{l}^{2}/3}+\sqrt{\alpha_{h}^{2}+\alpha_{l}^{2}/3}+\left(1-\alpha_{h}\right)/3\right]\left(r_{2}^{2}-r_{1}^{2}\right)}{\left(1-\alpha_{h}\right){\left(\alpha_{l}h_{2}\right)}^{2}}$
Type D	$\rho =\frac{4\sqrt{3}\pi \left[\sqrt{1+\alpha_{l}^{2}/3}+\sqrt{\alpha_{h}^{2}+\alpha_{l}^{2}/3}+\alpha_{l}/2+(1-\alpha_{h})/3\right]\left(r_{2}^{2}-r_{1}^{2}\right)}{\left(1-\alpha_{h}\right){\left(\alpha_{l}h_{2}\right)}^{2}}$

**Table 2:** Fitting parameters of Type A cell analyses.

	$\;\alpha_{h\;}=0$	$\;\alpha_{h\;}=0.1$	$\;\alpha_{h\;}=0.2$	$\;\alpha_{h\;}=0.3$	$\;\alpha_{h\;}=0.4$	$\;\alpha_{h\;}=0.5$
*e*	2.661	2.621	2.521	2.403	2.288	2.174
*R*^2^	0.999	0.999	0.999	0.999	0.999	0.999

**Table 3:** Fitting parameters of Type B cell analyses.

	$\;\alpha_{h\;}=0$	$\;\alpha_{h\;}=0.1$	$\;\alpha_{h\;}=0.2$	$\;\alpha_{h\;}=0.3$	$\;\alpha_{h\;}=0.4$	$\;\alpha_{h\;}=0.5$
*e*	2.893	2.719	2.332	2.032	1.836	1.715
*R*^2^	0.999	0.999	0.999	0.998	0.997	0.997

**Table 4:** Fitting parameters of Type C cell analyses.

	$\;\alpha_{h\;}=0$	$\;\alpha_{h\;}=0.1$	$\;\alpha_{h\;}=0.2$	$\;\alpha_{h\;}=0.3$	$\;\alpha_{h\;}=0.4$	$\;\alpha_{h\;}=0.5$
*e*	1.405	1.397	1.368	1.333	1.300	1.271
*R*^2^	0.999	0.999	0.999	0.999	0.999	0.999

**Table 5:** Fitting parameters of Type D cell analyses.

	$\;\alpha_{h\;}=0$	$\;\alpha_{h\;}=0.1$	$\;\alpha_{h\;}=0.2$	$\;\alpha_{h\;}=0.3$	$\;\alpha_{h\;}=0.4$	$\;\alpha_{h\;}=0.5$
*e*	1.553	1.658	1.653	1.559	1.468	1.393
*R*^2^	0.999	0.999	0.999	0.999	0.999	0.999

**Table 6:** The mechanical parameters of four NPR lattice structures.

(MPa)	Type A	Type B	Type C	Type D
First elastic modulus	209.7	461.45	1089.2	1271.7
Yield stress	8.24	17.04	14.56	25.15

**Table 7:** The densification strain and plateau stress of four NPR structures.

	Type A	Type B	Type C	Type D
$\mathrm{Densification}\;\mathrm{strain}\;\varepsilon_{d}$	0.588	0.656	0.676	0.651
$\mathrm{Plateau}\;\mathrm{stress}\;\sigma_{pl}$ (MPa)	8.28	13.21	13.06	18.76
